# Two-phase treatment of patients with crossbite and tendency toward skeletal Class III malocclusion^*^

**DOI:** 10.1590/2176-9451.19.4.122-135.bbo

**Published:** 2014

**Authors:** Maria de Lourdes Machado Bayerl

**Affiliations:** 1 Specialist in Orthodontics and Facial Orthopedics, PUC/Minas. Specialist in Pediatric Dentistry, Federal University of Minas Gerais (UFMG). MBA in Health management, FGV. Certified by the Brazilian Board of Orthodontics and Facial Orthopedics (BBO).

**Keywords:** Angle Class III malocclusion, Palatal expansion technique, Maxilla

## Abstract

Angle Class III malocclusion is characterized by an inadequate anteroposterior dental relationship which may or may not be accompanied by skeletal changes. In general, patients are distressed by a significantly compromised facial aspect which, when associated with a deficient middle third, encourages patients to seek treatment. This article reports a two-phase treatment carried out in a female patient aged six years and six months with a tendency towards a Class III skeletal pattern. This case was presented to the Brazilian Board of Orthodontics and Facial Orthopedics (BBO). It is representative of the Discrepancy Index (DI) category, and fulfills part of the requirements for obtaining BBO Diploma.

## Abstract (pt)

A má oclusão de Classe III de Angle é caracterizada por uma relação dentária anteroposterior inadequada, que pode ou não estar acompanhada de alterações esqueléticas. Em geral, o aspecto facial fica bastante comprometido, principalmente quando associado à deficiência no terço médio, sendo esse, na maioria das vezes, o principal fator que motiva o paciente a procurar pelo tratamento ortodôntico. Este artigo relata o tratamento realizado em duas fases, de uma paciente do sexo feminino, com seis anos e seis meses de idade, com tendência a um padrão esquelético de Classe III. Este caso foi apresentado à diretoria do Board Brasileiro de Ortodontia e Ortopedia Facial (BBO), representando a categoria com índice de grau de complexidade (IGC) igual ou acima de dez pontos, como parte dos requisitos para obtenção do título de Diplomada pelo BBO.

## INTRODUCTION

This report describes the case of a healthy female patient presented for treatment at the age of six years and six months. Her medical and dental history was within the normal range, with no history of illness or trauma. She had good oral hygiene and saw her pedodontist every six months. Her parents' chief complaint was that her upper teeth were positioned behind her lower teeth. The patient's father had Angle Class III malocclusion with a skeletal Class III pattern as well. Given that his daughter's dental arch resembled his own, he wanted her treatment to start as early as possible since he had been informed that in so doing she might avoid future surgery, or at least minimize it in case it was needed in adulthood.

## DIAGNOSIS

**Figure 1:**
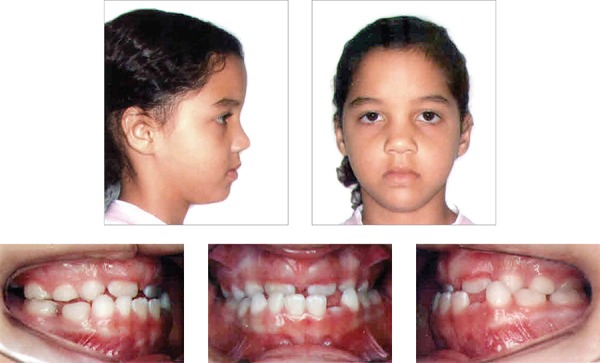
Initial facial and intraoral photographs.

**Figure 2:**
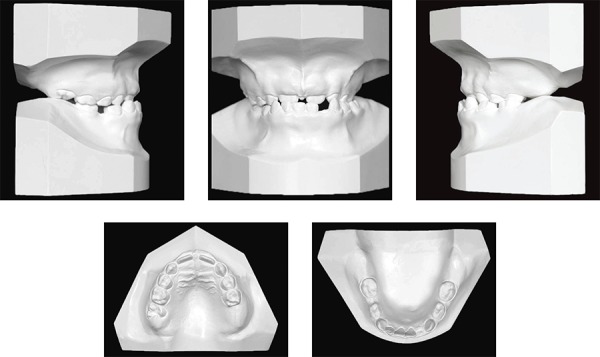
Initial casts.

Regarding her facial appearance, as shown in Figure 1, the patient had good symmetry, slightly convex profile, lip competence, slightly protruding lower lip and little exposure of upper incisors at speaking and smiling. Dental examination (Figs 1 and 2) revealed that the patient was in early mixed dentition with distal relationship of the primary molars in straight terminal plane while in the posterior region only tooth #16 had begun erupting. She presented with anteroposterior disharmony between maxilla and mandible, and maxillary constriction (atresia), resulting in a crossbite that extended from tooth #53 to #65. She also had a deep curve of Spee, 3.3 mm overbite and 4 mm negative overjet. A decrease in eruption spaces was also noted in teeth #12 and #22. The lower arch midline exhibited a 3.2 mm deviation, and there were spaces in the lower arch which produced a positive 5-mm discrepancy.

**Figure 3:**
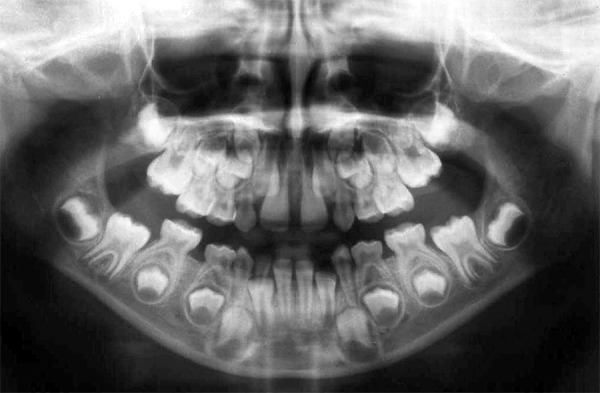
Initial panoramic radiograph.

**Figure 4:**
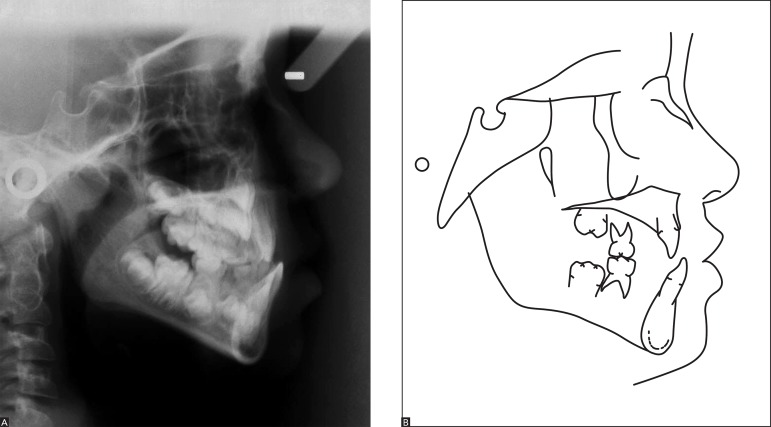
Initial profile cephalometric radiograph (**A**) and cephalometric tracing (**B**).

**Figure 5:**
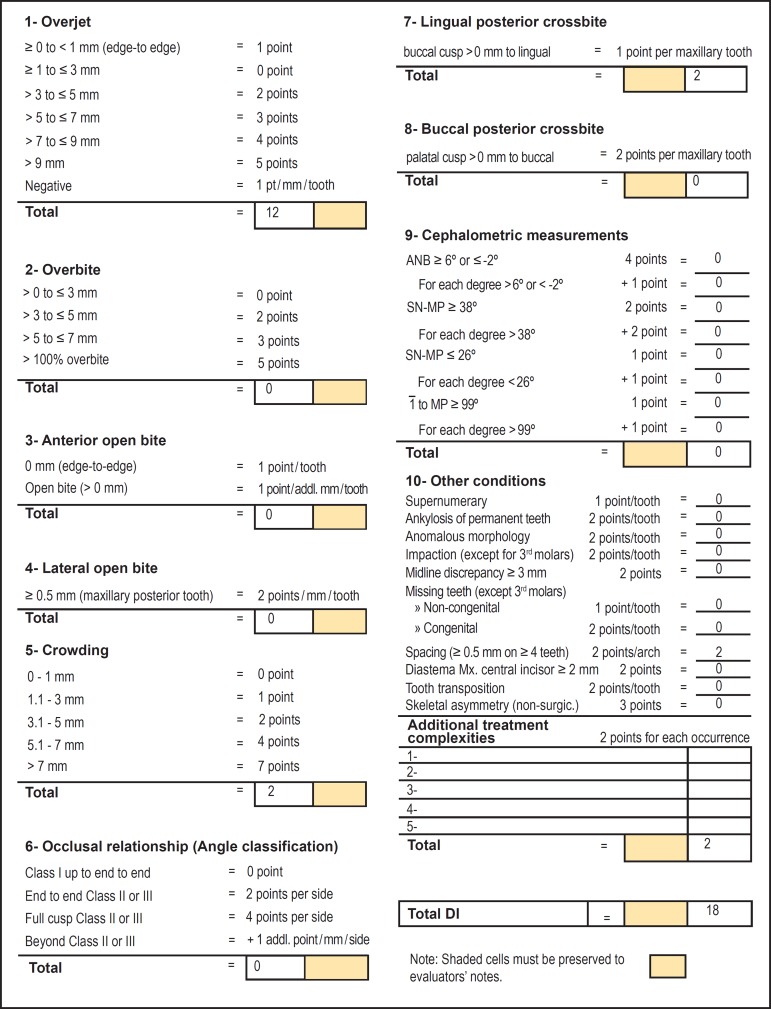
Calculation of the Discrepancy Index (DI) adapted by BBO.

In analyzing the panoramic radiograph (Fig 3), it was observed that all permanent teeth were present, indicting that bone and tooth structures were within normal limits. Cephalometrically (Fig 4 and Tab 1), although the value of the ANB angle was within normal range (ANB = 3.5°), it could be verified through Wits analysis (Wits = -3 mm) that the patient had a skeletal pattern tending towards Class III. This fact, coupled with her unfavorable family history - given that the patient's father had a dental and skeletal Class III pattern - reinforced the need for early intervention. A growth pattern with vertical tendency (SN-GoGn = 36°), severe axial inclination of upper incisors towards palatal (1-NA = 0°), and well positioned lower incisors (NB-1 = 26°) were also observed. It is noteworthy that mandibular manipulation caused no change from centric relation to maximum and habitual intercuspation (MHI). In calculating the Discrepancy Index (DI) a total of 18 points (Fig 5) was found.

## TREATMENT PLAN (PHASE 1)

Planning involved a two-phase treatment. Initially, special attention would be given to the skeletal disharmony between maxilla and mandible which, if left uncorrected, would contribute significantly to the worsening of this condition during the whole process of growth and development of the bony bases. Thus, the anterior and posterior crossbites would be corrected, enlarging the maxilla in the transverse direction, thereby gaining space in the anterior region for proper eruption of teeth #12 and #22.

The patient and her parents would then be informed of the importance of compliance in wearing the appliances and the need to perform treatment in two phases. In this first phase, the patient was six years and six months old. The posterior crossbite would be corrected using a modified Haas palate expansion appliance supported on the second primary molars with acrylic covering the incisor surface of primary canines and occlusal surfaces of primary molars . Additionally, hooks would be soldered to the buccal surface of orthodontic bands fitted on second primary molars, combined with finger springs to procline teeth #11 and #21.

Maxillary protraction would be performed (a) to correct the anteroposterior discrepancy, and (b) to procline the incisors with finger springs. This procedure would make use of Hickham's chin cup. After this stage, the patient would be monitored every six months until the ideal moment came to implement the second treatment phase.

At this stage, no orthodontic appliance was placed in the patient's lower dental arch.

## TREATMENT PROGRESS

The modified Haas appliance was installed and the patient was instructed to make 1/4 turn activations every 12 hours for 15 days. After this period, the expander was stabilized and the patient started to wear Hickham's chin cup with a force of 400 g on each side for 16 hours/day for 11 months. After this period, the modified Haas appliance was removed and the patient began being evaluated every six months.

## RESULTS

**Figure 6:**
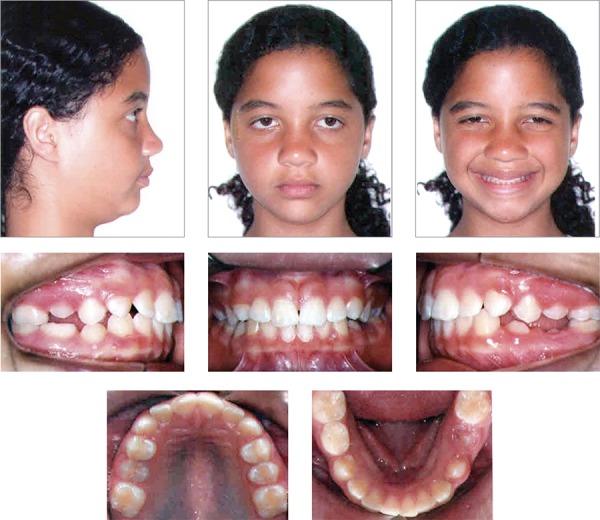
Intermediate facial and intraoral photographs.

**Figure 7:**
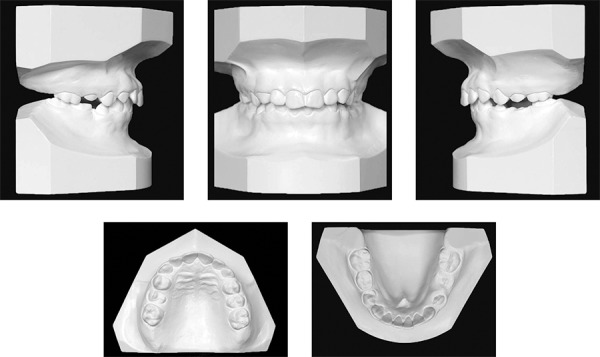
Intermediate cast models.

**Figure 8:**
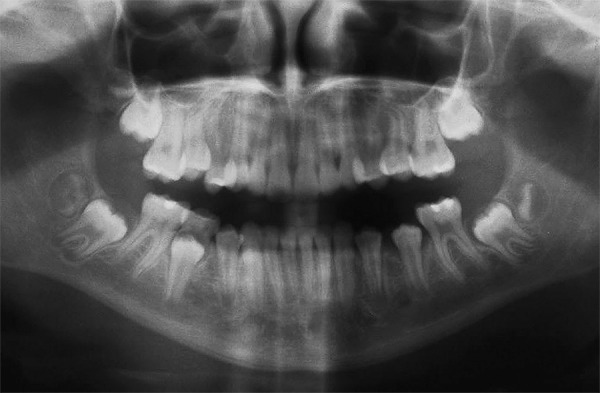
Intermediate panoramic radiograph.

**Figure 9:**
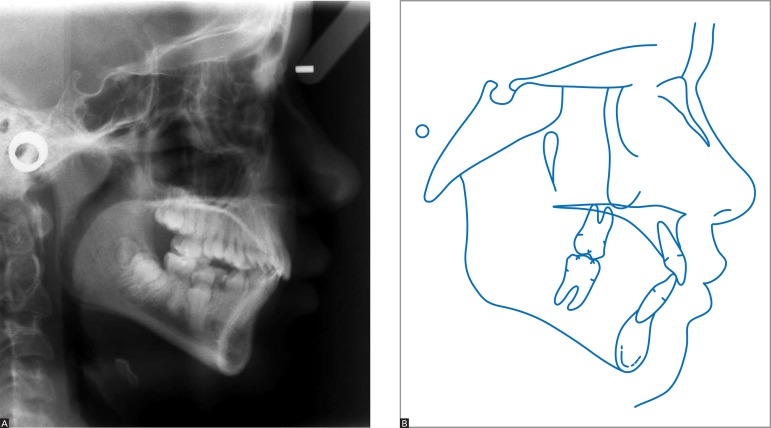
Intermediate profile cephalometric radiograph (**A**) and cephalometric tracing (**B**).

**Figure 10:**
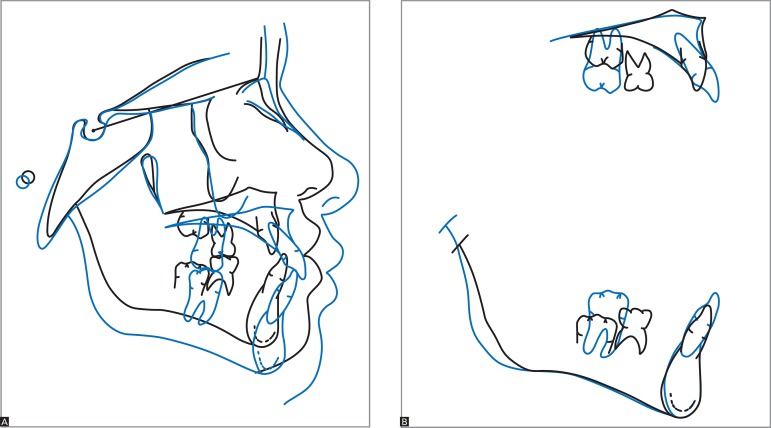
Total (**A**) and partial (**B**) superimposition of initial (black) and intermediate (blue) cephalometric tracings.

After analysis of the examinations carried out in planning the second treatment phase, it was observed that the objectives proposed for the first phase had been fully accomplished (Figs 6 to 10 and Tab 1). From a dental perspective, anterior and posterior crossbites were corrected, and consequently overbite and overjet were also resolved, thus improving alignment. As planned, a pattern of Class II molar relationship was attained through forward displacement of the maxilla, and sufficient space for proper eruption of teeth #12 and #22.

Cephalometrically, there was an increase in the ANB angle to 5° (SNA = 82° and SNB = 77°) and a significant improvement in Wits value (Wits = 0 mm). This change was especially due to displacement of the maxilla forward and downward, although there was a slight counterclockwise rotation of the mandible (SN-GoGn increased from 36° to 33° and FMA went from 21° to 17°).

## TREATMENT PLAN (PHASE 2)

After four years monitoring patient's development with appointments every six months, the second treatment phase started during the period of pubertal growth spurt, when the patient was 11 years and 10 months old. The goals in this phase were to align and level the teeth, achieving a perfect molar relationship and ultimately finishing the treatment. After finishing, extraction of third molars would be indicated. To this end, a preadjusted fixed orthodontic appliance, Roth prescription, 0.022 x 0.028-in slot would be bonded to upper and lower dental arches. In the finishing stage, individualized bends would be placed as required in stainless steel, 0.018 x 0.025-in archwires, and intermaxillary elastics would be prescribed. After finishing, the retention phase would begin with the use of a wraparound removable appliance placed in the upper arch and a lingual canine-to-canine retainer in the lower arch.

## TREATMENT PROGRESS

The upper arch received orthodontic bands with convertible triple tubes (0.051-in) welded to them, placed on teeth #16 and #26. Thereafter, preadjusted metal brackets (Roth prescription, 0.022 x 0.028-in slot) were bonded to upper incisors for alignment and leveling with the aid of Twistflex 0.018-in wire and stainless steel 0.016-in and 0.018-in wire, with expansion loop. After slight flaring of incisors, brackets were bonded to upper canines and premolars.

As of his moment, straight archwires made with 0.016-in, 0.018-in and 0.020-in were used. Eighteen months into treatment, teeth #17 and #27 erupted and were included in alignment and leveling of teeth. Upon finishing, as planned, rectangular 0.018 x 0.025-in stainless steel archwires were formed, torqued and individually coordinated as needed.

Orthodontic bands with convertible rectangular double tubes were fitted to teeth #36 and #46 in the lower arch, and 20 months into treatment, brackets were bonded to teeth #37 and #47. Alignment and leveling was performed using 0.018-in twist-flex archwires and round 0.016-in, 0.018-in and 0.020-in stainless steel archwires. The case was finished with 0.018 x 0.025-in rectangular stainless steel archwires with form, torques and coordination performed as needed on an individual basis. Class II intermaxillary elastics in Class II direction were also employed to decrease lower anchorage and thus achieve a perfect molar relationship. After performing this step, positions of maximum habitual intercuspation (MHI), centric relation and functional guidances (right and left laterality, and protrusion) were verified.

After ensuring that all planned objectives had been achieved, upper and lower fixed orthodontic appliances were removed and the retention phase began. In this phase, a removable wraparound appliance was fitted in the upper dental arch with a buccal 0.032-in stainless steel arch. The patient was instructed to wear it 24/7 for 18 months. After this period, she would wear it 12 hours a day for six months, then only at nighttime for another six months. At the end of this period, the case would be assessed to determine whether or not to suspend its use altogether. In the lower arch, a round 0.018-in stainless steel fixed canine-to-canine retainer was bonded permanently to the lingual surfaces of teeth #33 through #43.

## RESULTS

**Figure 11:**
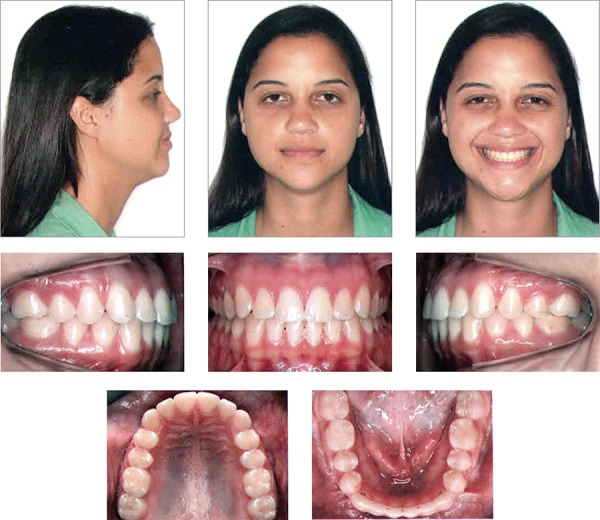
Final facial and intraoral photographs.

**Figure 12:**
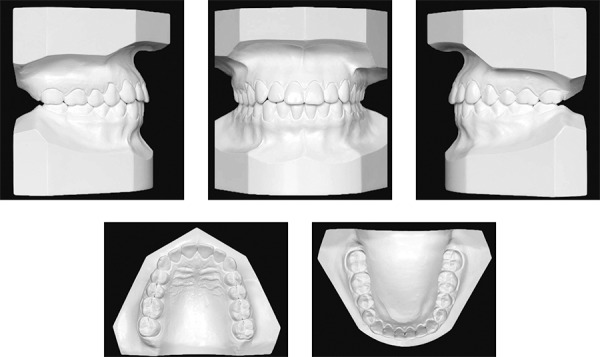
Final dental casts.

**Figure 13:**
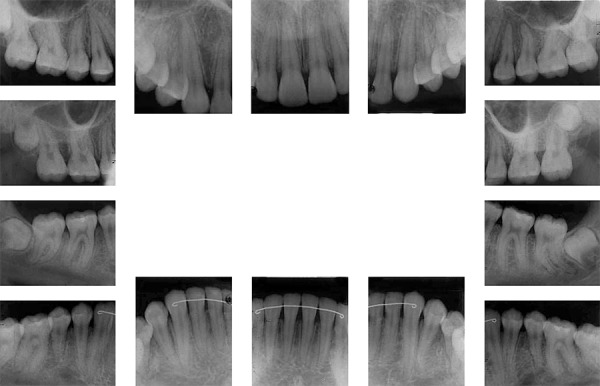
Final periapical radiographs.

**Figure 14:**
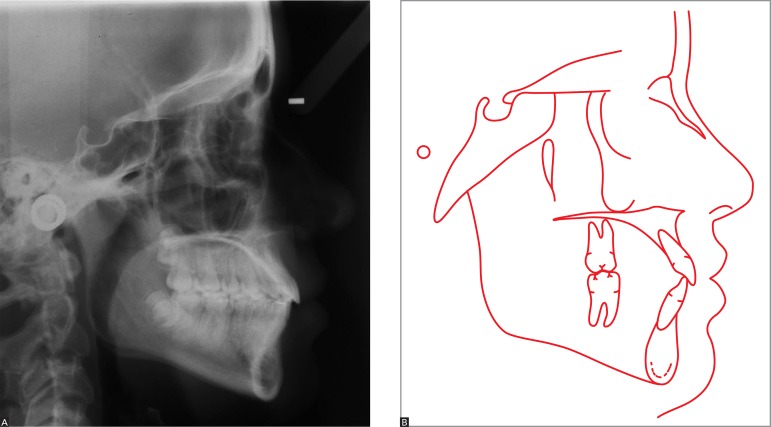
Final profile cephalometric radiograph (**A**) and cephalometric tracing (**B**).

At the end of treatment, additional exams were required (Figs 11 to 14). They revealed that results were very favorable as all objectives were clearly achieved. Concerning the facial features, as shown in Figure 11, the smile exhibits an adequate exposure of upper incisors, and the lower lip is parallel to the upper teeth, displaying an excellent smile arc. Balanced facial symmetry and lip competence can also be observed. In analyzing the profile, it appears that despite an overall harmony, the mandible still shows slight protrusion.

In examining the teeth (Figs 11 and 12), it was observed that proper alignment and leveling were achieved, as well as proper occlusion with a perfect relationship between upper and lower molars, and between the upper and lower canines, on both sides. Overbite, overjet, posterior crossbite and lower midline were corrected. The spaces in the lower dental arch were closed and proper intercuspation obtained. There was an increase in intercanine and intermolar widths in both dental arches. In the upper arch, the intercanine width was 25 mm to 36 mm, and intermolar width 41 mm to 51 mm. In the lower arch, the intercanine width was 20 mm to 29 mm, and intermolar width 39 mm to 44 mm. Functional harmony was attained in protrusive position, as well as right and left laterality and the centric relation coincided with the intercuspid position. It is noteworthy that all these results were achieved with only mild apical remodeling. No sign of significant root resorption was observed at the end of treatment (Fig 13).

As expected, several skeletal changes were observed. Mandibular growth occurred in the anterior direction, which caused a decrease in the ANB angle from 3.5° to 1° while the SNA angle remained unchanged and the SNB angle rose from 77.5° to 80°. The vertical dimension was controlled, with a significant decrease in the mandibular plane angle (SN-GoGn changed from 36° to 29° and FMA from 21° to 17°). These data can be viewed in Figure 14 and Table 1.

**Table 1:** Initial (A), intermediate (A1) and final (B) cephalometric values.

	Measurements		Normal	A	A1	B	A/B diff.
**Skeletal pattern**	SNA	(Steiner)	82°	81°	82°	81°	0
	SNB	(Steiner)	80°	77.5°	77°	80°	2.5
	ANB	(Steiner)	2°	3.5°	5°	1°	2.5
	Angle of convexity	(Downs)	0°	8°	13°	1°	7
	Axis Y	(Downs)	59°	52°	55°	54.5°	2.5
	Facial angle	(Downs)	87°	93.5°	92°	95°	1.5
	SN-MP	(Steiner)	32°	36°	33°	29°	7
	FMA	(Tweed)	25°	21°	17°	17°	4
**Dental pattern**	1 to MP	(Tweed)	90°	93°	99°	94.5°	1.5
	1.NA (degrees)	(Steiner)	22°	0°	20°	29°	29
	1-NA (mm)	(Steiner)	4 mm	0 mm	4 mm	8 mm	8
	1.NB (degrees)	(Steiner)	25°	26°	30°	25°	1
	1-NB (mm)	(Steiner)	4 mm	4 mm	7 mm	5 mm	1
	^1^⁄_1_ - Interincisal angle	(Downs)	130°	151°	125°	126°	25
	1-APo	(Ricketts)	1 mm	4 mm	4 mm	4 mm	0
**Profile**	Upper lip — S line	(Steiner)	0 mm	2 mm	2 mm	0 mm	2
	Lower lip — S line	(Steiner)	0 mm	3.5 mm	2.5 mm	1 mm	2.5

Cephalometric total superimpositions revealed that the maxilla and mandible moved forward and downward. Partial maxillary superimpositions during the first phase showed vertical development of the alveolar process in the molars, and a substantial change in the inclination of upper incisors. In the second phase, molar mesialization and extrusion were observed along with mild flaring and extrusion of incisors. Partial mandibular superimpositions during the first phase showed mesialization and extrusion of molars, with flaring and extrusion of incisors. In the second phase, molar extrusion and incisor uprighting were noted.

## FINAL CONSIDERATIONS

Diagnosis and treatment planning of Class III malocclusion should be performed judiciously. Moreover, patients and their families should be made aware of the entire process as results can be unpredictable. Even if goals and a success rate have been established, the prognosis is unclear, since it depends largely on case development and patient compliance. Even so, it is likely that once patient's growth has ended the need for retreatment by dental compensation^1^ or surgical intervention may still exist.^2^

No consensus has yet been reached in the literature regarding the ideal time to start treatment since some authors posit that growth and development of the craniofacial complex is genetically determined, and therefore unalterable. For these authors, correction of most Class III cases necessarily involves surgery. Furthermore, orthodontic treatment should be started immediately after the growth spurt period has ceased. Other authors however - although agreeing with the role of heredity in the etiology of Class III - believe they can change growth pattern and direction by means of a non-surgical approach capable of minimizing the malocclusion or even treating it successfully.^2^ When the patient is in the phase that precedes the pubertal growth spurt, early treatment is indicated given that Class III malocclusions tend to become increasingly severe over time; the reason being that mandibular growth remains active for a longer period than maxillary growth.^2,3^ Treatment of the case reported here was conducted in two phases. It was initiated when the patient was six years and six months old. Younger individuals tend to achieve more favorable results between four and ten years of age, although patients whose ages range from 10 to 14 years old also present positive results.^3,4^

In the first phase, rapid maxillary expansion was combined with maxillary protraction using Hickham's^5^ chin cup. This device is easy to fabricate and individualize. It has met with wide acceptance by patients, besides being more stable, especially during sleep.^6^ Although there are several types of appliances for orthopedic protraction of the maxilla, the literature contains no studies comparing their efficiency. The skill of the professional handling the device and patient comfort are important variables when choosing one of these appliances.^2,7,8^

The second treatment phase began in late mixed dentition. At this stage, there was large overcorrection, and the patient was in Angle Class II malocclusion. Furthermore, the mandible was slightly retruded, which is indicated in this case since she would otherwise continue to present with an unfavorable growth pattern.^8^ At this stage, a conventional fixed orthodontic appliance was used. Treatment outcome was very favorable, both functionally and esthetically. Upper arch expansion, which had been performed in the first phase, was stable, indicating that the morphological limits were respected. Dental alignment and leveling were achieved in the second stage. Canine and molar occlusion was well established and anterior and posterior crossbites corrected,^9^ with pleasing facial esthetics, good symmetry and adequate exposure of the upper incisors.

## Figures and Tables

**Figure 15:**
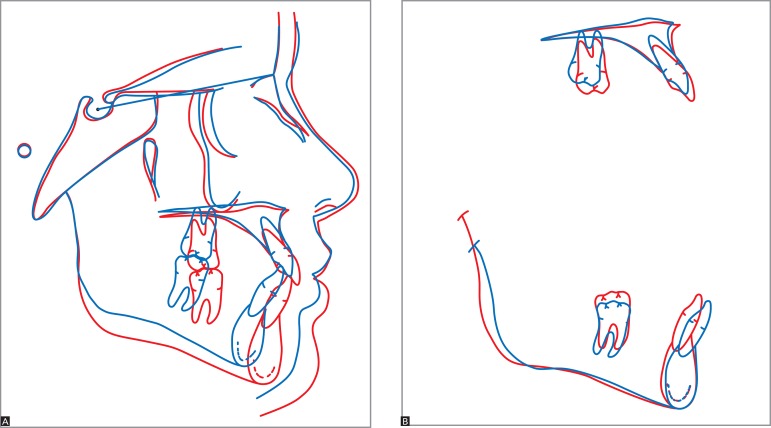
Total (**A**) and partial (**B**) superimpositions of intermediate (blue) and final (red) cephalometric tracings.

**Figure 16:**
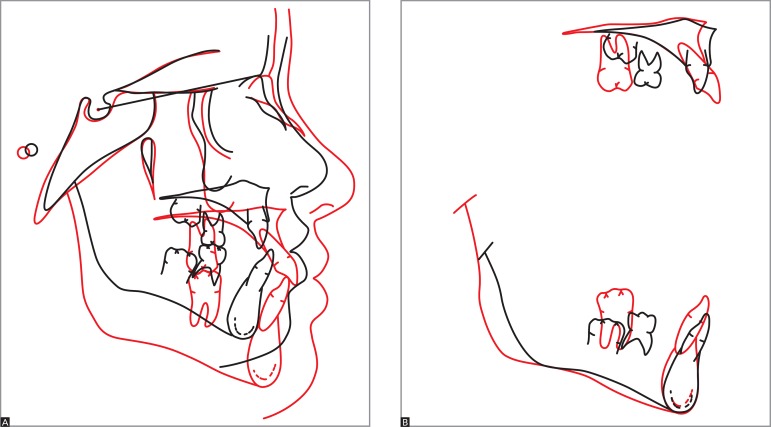
Total (**A**) and partial (**B**) superimpositions of initial (black) and final (red) cephalometric tracings.
